# Gait analysis methods in rehabilitation

**DOI:** 10.1186/1743-0003-3-4

**Published:** 2006-03-02

**Authors:** Richard Baker

**Affiliations:** 1 Hugh Williamson Gait Analysis Service, Royal Children's Hospital, Parkville, Victoria, Australia; 2 Gait CCRE, Murdoch Children's Research Institute, Parkville, Victoria, Australia; 3 Department of Mechanical and Manufacturing Engineering, University of Melbourne, Parkville, Australia; 4 Musculoskeletal Research Centre, La Trobe University, Bundoora, Victoria, Australia

## Abstract

**Introduction:**

Brand's four reasons for clinical tests and his analysis of the characteristics of valid biomechanical tests for use in orthopaedics are taken as a basis for determining what methodologies are required for gait analysis in a clinical rehabilitation context.

**Measurement methods in clinical gait analysis:**

The state of the art of optical systems capable of measuring the positions of retro-reflective markers placed on the skin is sufficiently advanced that they are probably no longer a significant source of error in clinical gait analysis. Determining the anthropometry of the subject and compensating for soft tissue movement in relation to the under-lying bones are now the principal problems. Techniques for using functional tests to determine joint centres and axes of rotation are starting to be used successfully. Probably the last great challenge for optical systems is in using computational techniques to compensate for soft tissue measurements. In the long term future it is possible that direct imaging of bones and joints in three dimensions (using MRI or fluoroscopy) may replace marker based systems.

**Methods for interpreting gait analysis data:**

There is still not an accepted general theory of why we walk the way we do. In the absence of this, many explanations of walking address the mechanisms by which specific movements are achieved by particular muscles. A whole new methodology is developing to determine the functions of individual muscles. This needs further development and validation. A particular requirement is for subject specific models incorporating 3-dimensional imaging data of the musculo-skeletal anatomy with kinematic and kinetic data.

**Methods for understanding the effects of intervention:**

Clinical gait analysis is extremely limited if it does not allow clinicians to choose between alternative possible interventions or to predict outcomes. This can be achieved either by rigorously planned clinical trials or using theoretical models. The evidence base is generally poor partly because of the limited number of prospective clinical trials that have been completed and more such studies are essential. Very recent work has started to show the potential of using models of the mechanisms by which people with pathology walk in order to simulate different potential interventions. The development of these models offers considerable promise for new clinical applications of gait analysis.

## Introduction

For the purposes of this paper *gait analysis *will be assumed to refer to the instrumented measurement of the movement patterns that make up walking and the associated interpretation of these. The core of most contemporary gait analysis is the measurement of joint kinematics and kinetics. Other measurements regularly made are electromyography (EMG), oxygen consumption and foot pressures. A systematic physical examination of the patient is usually conducted as part of a gait analysis.

Rehabilitation is a clinical discipline and this paper will thus concentrate on *clinical *gait analysis. Richard Brand [1,2] proposed four reasons for performing any clinical test (see Table 1). The third of these might actually be taken as a definition of the word *clinical *i.e. a *clinical *test is one conducted in order to select from among different management options for a patient (including the possibility of not intervening).

**Table 1:** Reasons performing clinical tests as stated by Brand [1, 2])

1. to distinguish Diagnosis between disease entities (diagnosis).
2. to determine severity of disease or in jury (i.e. assessment or evaluation)
3. to select among treatment options
4. to predict prognosis

Much contemporary gait analysis is done for the purpose of *clinical research*. This differs from clinical testing in that the reason is not to make clinical decisions for the individual patient, but to learn about a condition affecting a group of patients or the effect of an intervention. It is important to remember that the criteria for valid clinical research may not be the same as those for valid clinical testing. For example if a measurement made on a patient cannot be relied upon because of random errors then that measurement will not be useful for clinical purposes. By increasing the number of patients in a sample however, even measurements with quite large random errors can result in meaningful conclusions in clinical research. This paper will focus on gait analysis for clinical use. It will also focus on methodology rather than areas of clinical application.

Brand's [1,2] other three possible reasons for performing any clinical test are to distinguish between disease entities (diagnosis), to determine the severity, extent or nature of a disease or injury (assessment), and to predict outcomes of intervention (or the absence of intervention). The monitoring of the progress of a patient's condition either following intervention or in its absence might be regarded as an additional reason. This modification of Brand's approach is summarised in Table 2.

**Table 2:** Reasons performing clinical gait analysis (modified from Brand [1, 2])

Clinical gait analysis is performed to allow the selection from amongst treatment options (including the possibility of not intervening). This is based on one or more of:
1. *Diagnosis *between disease entities.
2. *Assessment *of the severity, extent or nature of a disease or injury.
3. *Monitoring *progress in the presence or absence of intervention.
4. *Prediction *of the outcome of intervention (or the absence of intervention).

Brand went on to propose a number of criteria for assessing the usefulness of biomechanical measurements in general which, with some modification, can be used as criteria for the usefulness of all clinical gait analysis. These are listed in Table 3. The first requirement of any clinical measurement is that it should characterise the patient, that is if the patient attends on two separate occasions, between which his or her condition might be considered as stable, the measurements taken should be similar. This requires that the measurement technique itself is repeatable but also that the quantity being measured is stable and independent of factors such as mood, motivation or pain. Measurements can be repeatable and stable without necessarily being accurate (representative of a specific physical quantity). Such tests can be clinically useful but will be much easier to interpret if they are also accurate. In an era of evidence based clinical practice it is essential that any measurement techniques are appropriately validated which must include assessments of both their repeatability and accuracy.

**Table 3:** Criteria for biomechanical measures (extracted from text of Brand [1])

Reproducible
Stable (independent of mood, motivation and pain)
Accurate
Appropriately validated
Capable of distinguishing between normal and abnormal
Must not alter the function it is measuring
Reported in form analogous to accepted clinical concepts
Cost-effective
Not observable by the skilled clinician

In order to perform a diagnostic function it is necessary for measurements to be able to distinguish normal from abnormal patterns of movement and also between the characteristics of one disease entity and another. There are two aspects to this. The first is having measurement systems capable of working to adequate precision. The second is a knowledge of what characterises normal walking or a particular disease entity.

The requirement for patient assessment pre-supposes that a diagnosis does not give sufficient information to determine the most appropriate management for a patient and that measuring the precise characteristics of a patient's condition are essential for this. Measurements thus have to be sufficiently precise to reveal clinically important differences between patients with the same diagnosis. For monitoring purposes measurements need to be sufficiently precise to be able to determine whether a patient's condition is stable, improving or deteriorating.

Brand suggested that the measurement technique should not affect the function it is measuring. The walking performed in a gait analysis laboratory however, with the patient concentrating on what they are doing in an idealised environment, is not necessarily representative of their normal walking. At the very least this must be taken into account when interpreting results.

Gait analysis should reveal information that is useful to the clinician and this will generally require that results are reported in terms analogous to accepted clinical concepts. It must be cost-effective, that is the benefit of performing the test must be worth the cost. This balance need not necessarily be determined in purely financial terms but the financial cost of gait analysis is a significant factor. Finally there is no point doing any clinical test if the results could be obtained sufficiently well by simply observing the patient

The information obtained by assessing the patient is that used for selecting management options. This process does not, therefore, make further demands on the measurement systems but does require an understanding of how the patient's condition is likely to be affected by an intervention (or none) to a level sufficient to determine which options are preferable. Prediction of outcomes takes this one stage further to being able to determine not only which management option is best but also how the patient will be after that intervention.

This sequential analysis of the four potential purposes of clinical tests reveals a progression from just requiring reliable and precise measurements to the additional requirement of having an understanding of how such information is incorporated into clinical practice. The state of the art is that the measurement component of gait analysis can reasonably be described as an objective process whereas the interpretation component is predominantly subjective.

Making the interpretive component more objective can be achieved in two ways. The first is to develop a general theory of how people walk whether they have recognised pathology or not. As long ago as 1982 Cappozzo lamented, "The approaches to clinical gait analysis and evaluation are not supported by general theories" [3] and despite over 20 years of intense activity this is still a reasonable summary of the state of the art. The second approach, which must operate in the absence of the former, is to conduct clinical research to ascertain the outcome of particular interventions on groups of patients characterised by certain measurements. Most of the knowledge base used in the interpretive component of gait analysis comes from such studies. It is because there are relatively few studies available to base such interpretations on that the subjective element of interpretation is necessary in contemporary clinical gait analysis.

## Measurement methods in clinical gait analysis

Modern clinical gait analysis traces its origins back to the early 1980s with the opening of the laboratory developed by the United Technologies Corporation at Newington, Connecticut and those provided with equipment by Oxford Dynamics (later to become Oxford Metrics) in Boston, Glasgow and Dundee. Retro-reflective markers were placed on the skin in relation to bony landmarks. These were illuminated stroboscopically and detected by modified video cameras. If two or more cameras detect a marker and the position and orientation of these cameras are known then it is possible to detect the three-dimensional position of that marker [4].

Whilst the basic principles remain the same as the earliest systems, the speed, accuracy and reliability has advanced beyond all recognition. It is not uncommon now to find clinical systems using 8, 10 or more cameras functioning at over 100 Hz and capable of detecting reliably the presence of many tens of markers of between 9 and 25 mm diameter. Calibration of the systems (the determination of the position, orientation and optical and electronic characteristics of the cameras) can generally be accomplished in less than a minute. Marker positions from clinical trials can be reconstructed and markers labelled automatically in real time (although this feature is often not essential for clinical studies). The determination of the accuracy of such systems is now generally limited by the accuracy of any alternative means to determine marker position and can be taken to be of the order of 1 mm. This is probably an order of magnitude smaller than other sources of error in determining joint kinematics and kinetics. This particular measurement technology has thus reached a mature state of development that, whilst advances will almost certainly continue, already probably delivers all that is required by conventional gait analysis [5].

The same cannot be said of the computer models used to derive joint kinematics and kinetics from the marker position data supplied by the measurement hardware. Almost all commercially available clinical systems use some variant of the Conventional Gait Model [6] which has been referred to as the Newington, Gage, Davis [7], Helen Hayes, Kadaba [8,9] or Vicon Clinical Manager (VCM) model. This was developed using the minimum number of markers possible to determine 3-dimensional kinematics and kinetics [10,11] of the lower limb at a time when measurement systems were only capable of detecting a handful of markers. It assumes three degree of freedom joints for the hip and knee and a two degree of freedom joint at the ankle. The model is hierarchical requiring the proximal segments to have been detected in order that distal segments can be defined and incorporates regression equations to determine the position of the hip joint centre with respect to pelvic markers. Kinetics are determined using an inverse dynamics approach which generally requires considerable filtering to give any useful signals. An alternative system the Cleveland Clinic Model based around a cluster of markers on a rigid base attached to each segment is the only other widely used model. Unfortunately documentation of this model in the scientific literature is very poor.

### The problem of limited repeatability

The primary problem of current measurement technology is that of reliability in routine clinical use. Several studies have now been reported in which a single subject has been analysed in a number of different laboratories [12-14]. These have shown a degree of variability between sites that would appear to be sufficient to undermine clinical applications. In retrospect, the original studies of the reliability are flawed. There was no such study of the Davis implementation of the model and the statistics used by Kadaba et al [8,9] to report reliability of their implementation probably acted to mask deficiencies. In particular, use of relative measures of reliability such as the coefficient of multiple correlation (CMC) makes interpretation of findings difficult. Almost all reliability studies have been done on subjects without pathology where marker placement is reasonably straightforward. Reliability for clinical populations is rarely reported in the literature and is almost certainly inferior.

At least one recent study has shown that it is possible to get levels of reliability sufficient to justify the continued clinical use of gait analysis within a single centre [15]. Too few centres however are providing evidence to establish that this is the rule rather than the exception.

**Whilst not the most exciting field of research, a very real need of clinical gait analysis is for the development of techniques for establishing the reliability of measurement techniques and of methods of quality assurance that will ensure that the very highest standards of reliability are achieved in routine clinical practice**.

### Source of error: Model calibration

There are two principal sources of error. The first is the difficulty determining the anthropometry of the individual subject (known as *model calibration*). This has two aspects, placing markers accurately with respect to specific anatomical landmarks and determining the location of the joint centres (and other anatomical features) in relation to these markers. Failure to place markers accurately is probably the single greatest contributor to measurement variability in contemporary clinical gait analysis. This is partly a matter of appropriate staff training and quality assurance but at least as important, and more fundamental, is the problem that many of the landmarks used to guide marker placement are not themselves particularly well defined in patients with certain conditions [16]. Even when bony landmarks are sharply defined an increasing number of patients have a considerable thickness of subcutaneous fat that makes palpation difficult.

The Conventional Gait Model uses regression equations to determine the position of the hip joint centre in relation to the pelvis. Both Bell's [17-19] and Davis' [7] equations are commonly used and there is now good evidence that neither is satisfactory in healthy adults [20]. There have still been no published studies of whether either is valid for healthy children. Children with orthopaedic conditions including cerebral palsy may often have dysplasia of the hip or deformity of the pelvis, and it is exceedingly unlikely that any form of regression equation could be used in these patients to determine hip joint position.

Methods for moving away from anatomical landmarks and regressions equations for determining joint centres have been around for nearly a decade, the process being known as *anatomical calibration *[21]. They rely on calibration movements to be performed before capturing walking data and some form of fitting of the measured marker positions to an underlying model of how the body moves. The simplest example is probably the determination of the hip joint centre. It is assumed that the hip joint moves as a ball and socket joint about centre of rotation fixed in the pelvis. Any marker on the femur would thus be expected to describe a path on the surface of a sphere centred on the hip joint centre when the hip joint is moving. A least squares fit of the measured data to such a sphere allows the location of that joint centre to be determined [20,22]. Similar approaches are applicable to determine that axis of the knee joint which for this purpose has often been assumed to be a simple hinge joint.

Various approaches to fitting data to an underlying model have been attempted and many seem to give reasonable results [20,22-28]. Such techniques have not so far been widely accepted into clinical practice probably because there is a perception that such calibration trials are too difficult for patients to execute. At least one clinical lab however has now committed itself to implementing such techniques into routine practice and has reported failure to perform test adequately in only one of over 700 patients tested so far.

**Further studies are needed to confirm these studies and to identify which of the range of available optimisation techniques is the best suited to clinical applications. Comprehensive reliability studies are again needed to demonstrate the advantages of using such models over the conventional model**.

### Sources of error: Soft tissue artefact

The second source of error is the degree of movement of the skin, muscle and other soft tissues in relation to the bones that occurs during walking. This is perhaps most marked in relation to the rotational profile of the hip. Lamoreux [29], as far back as 1991, reported that with optimal placement of thigh wands only 65% of transverse plane hip joint rotation was detected and that with poor placement this could be as little as 35%.

The problem of skin and other soft tissue movement is more problematic than that of model calibration. Lu and O'Connor where the first to propose *fitting *a model of how the body is expected to move to marker co-ordinate data [30] using an optimisation approach. This model uses a least squares fit, similar to some of the techniques described above for model calibration, and thus makes no assumptions about the nature of the soft tissue movement. Other similar models have now been made commercially available [26]. More recent studies have started to try to map out the movement of markers with respect to the underlying bones [31,32]. If such movement can be characterised as a function of joint angle then, in principle, this knowledge could be built into a model to allow such movements to be compensated for. Such mapping is only likely to be useful if it can be shown that soft tissue movement is consistent across a range of subjects and activities. It is not clear at present whether these conditions are satisfied. A particular problem in regard to mapping soft tissue movement is that of defining what the "true" movement of the bones is. In the absence of any gold standard a variety of assumptions are being used most of which have serious limitations.

**Significant work is needed in this area. A gold standard method for determining joint movement is required.**

**Maps of soft-tissue movement as a function of joint angle are required and work done to establish how these vary from individual to individual and from task to task.**

**Marker sets need to be defined based on the optimum placement of markers given knowledge of the soft-tissue displacements.**

**Finally it is possible that knowledge of likely soft-tissue displacement could be built into the optimisation algorithms allowing for better estimates of the movements of the underlying skeleton.**

The development of a gold standard method for determining joint movement will probably require a move away from skin-mounted markers (or other sensors). Once such technology is available however it is quite possible that this will supersede the presently available systems. The cost of any such new systems however is likely to prohibit ready clinical availability in the foreseeable future.

There has been some work done on markerless optical methods. By placing a number of video cameras around a subject and tracing the silhouette of the walking subject on each it is possible to generate a 3-dimentional silhouette of that subject. This has already been achieved but the next step of using such a silhouette to determine the co-ordinate systems associated with the moving body segments has not yet been satisfactorily achieved.

It is possible that the problem of skin movement can only be satisfactorily addressed by making direct measurements of bone position. It is now possible to take 3-dimensional images of bones (and muscles) using MRI but only within a very restricted capture volume [33-35]. The image processing problem of automatically determining a bone embedded axis system from such images has yet to be solved satisfactorily. Similarly both uniplanar and biplanar cine fluoroscopy [36-40] has been used to detect the 3-dimensional movement of the internal knee prostheses during a variety of movements. This is possible because a knowledge of the exact size and shape of the prosthetic components and their opacity to x-rays greatly simplifies the image processing problem. Using similar techniques to determine the movement of joints has also been reported [41-43]

**Using 3-dimensional imaging techniques to directly determine bone movements during walking either as a technology with potential clinical applicability or for use as a gold reference standard from which to improve the implementation of conventional marker based technologies is one of the greatest challenges in this area.**

## Methods for interpreting clinical gait analysis data

The second element of clinical gait analysis is the interpretation of data. Conventions for describing 3-dimensional joint kinematics and kinetics are well formulated. Many laboratories are augmenting conventional kinematics and kinetics with muscle length and, less commonly, moment arm graphs. Normal patterns of movement as represented by these data are now generally fairly well understood by clinical specialists although there is actually very little normative data published in the peer-reviewed literature. Similarly, many abnormal patterns of movement are quite widely recognised by clinicians but there few published attempts at formal classification of these [44-46]. Many clinicians have learnt to associate particular abnormal patterns in particular patient groups with particular impairments of body structure and function. Intervention based on such an understanding often leads to a normalisation of gait patterns at subsequent assessments (e.g. [47-55]). It is on this basis that clinical gait analysis operates at present.

Despite the widespread acceptance of many of these conventions there are still problems. Baker [56] demonstrated that the Euler sequence used to calculate pelvic angles gives rise to data that can be mis-leading to clinicians and proposed an alternative to correct this which is yet to be adopted widely within clinical analysis. Methods for interpreting angles in three dimensions, either in terms of Euler/Cardan rotations or the Grood and Suntay convention [57,58] are not well understood either by clinicians or many bioengineers. A recent attempt to standardise the reporting of joint angles [59] proposed a different convention to that of the Conventional Gait Model and the continuing debate as to which is preferable illustrates this confusion [60,61]. Joint moments are generally reported with reference to orthogonal axis systems fixed in the distal (Conventional Gait Model) or proximal segments (or occasionally the laboratory axis system). These differ significantly depending on the axis system chosen [6,62] yet there has been no debate about which if any is preferable. Reporting moments about orthogonal axis systems and joint rotations about non-orthogonal ones leads to difficulties in relating the moments to the changes in joint angles to which they are related. The use of muscle moment arms will be discussed further below but it is interesting that there is no straightforward definition of the meaning of the term *moment arm *in three dimensions [63] and it is often not clear how such data should be interpreted.

**A consistent, comprehensive and clear method for describing joint kinematics and kinetics in three dimensions would be of immense benefit for the clinical gait analysis community.**

Perhaps the most important limitation of our present understanding of human walking, however, is that it is primarily descriptive. We know *what *happens rather than *why *it happens. Many in the clinical gait analysis community regard kinematics as descriptive but contend that kinetics explain movement patterns. This is almost certainly misguided. Kinetics are simply another set of measurements and can thus only be descriptive.

There have been various attempts at establishing a theory of walking but none is particularly convincing. Saunders, Inman and Eberhart's determinants of normal walking [64] are perhaps the best known of these. Recent publications however have questioned how the detail of these reflects experimental data [65-70]. Gage [71,72] based his pre-requisites of gait on earlier work by Perry [73] but these are best regarded as pointers to where particular patients are deficient rather than explanations of how they are achieving walking with or without pathology.

Perhaps the closest we have come so far to understanding why we walk the way we do has come from the work of Pandy and Anderson [74,75]. They have shown that it is possible to construct a mathematical simulation of muscle function during normal walking based on the assumption that the total consumption of energy per unit distance walked is minimised. The authors, however, commented that the model seems more dependent on the boundary conditions imposed than on the nature of the optimisation function. Further, because of the complex nature of the optimisation process driving the model it is still difficult to explain how the precise characteristics of any particular feature of the walking pattern affect the overall calculation of energy expenditure. So far such a model has only been constructed for normal walking.

**An obvious challenge in the emerging field of computational biomechanics is to apply similar techniques to model walking with particular forms of pathology.**

Conceptually, modifying such models to incorporate a specific abnormality of the musculo-skeletal anatomy such as a leg length discrepancy or contracture of a particular muscle is reasonably straightforward. It is much less certain whether such techniques can be applied at all to patients with neuromuscular pathology who are most frequently seen by clinical gait analysis services. Optimisation techniques assume that movements are controlled in such a way that a specific control function is minimised. In many neuromuscular conditions (Cerebral Palsy, Parkinson's disease, adult hemiplegia) the problem is one of a loss of central control and this would appear to invalidate any techniques modelling human movement as an optimised process.

If such models are developed it will be interesting to see whether they give any insights into the clinical management of patients. Further it will be interesting to see whether their use leads to an understanding of why we walk the way we do which can be formulated as theories that are applicable without the use of such complex models.

**Perhaps the greatest challenge in clinical gait analysis is still to answer the question. "Why do we walk the way we do and why don't our patients?".**

Whilst the answer to this question still seems as far away as ever, significant advances have been made over recent years in understanding the mechanisms by which we walk particularly in the way that muscles act. For many years it was assumed that a muscle's anatomical position determines how it acts. It was assumed for example that the action of the hamstrings, passing behind the knee, was always to flex the knee. It is only comparatively recently that biomechanists have come to appreciate that any individual muscle has an effect on all the segments of the body and that in some circumstances this may result in a muscle having an action different to its anatomical function [76-81]. It is now fairly well accepted, for example, that the hamstrings functions as a knee extensor during early stance in normal walking because its effect in extending the hip has a secondary tendency to extend the knee which is greater than its direct effect as an anatomical knee flexor [82].

Such work depends on knowing the joint kinematics and kinetics and inertial properties of the body segments. These can be used to estimate the forces in individual muscles [81-83]. This is an indeterminate problem so is dependent on an optimisation approach (and the validity of this in neuromuscular pathology is questioned in the same way as that of the simulations described above). Once the muscle forces are known forward modelling can be used to determine the effect that a given muscle is having on any segment (or joint) of the body. Until very recently the first part of this problem, the estimation of muscle forces had not been achieved which limited the application of the second part, the forward modelling to data obtained from the simulations described above [74,75]. Recently methods have been develop to estimate the muscle forces required to generate measured joint kinematics and ground reaction forces and have been used both to understand the function of individual muscles during pathological gait and predict the effect of interventions [84,85]. These have been based on scaled models of the adult musculo-skeletal anatomy.

**A further area of challenge is in using 3-dimensional imaging techniques to model musculo-skeletal deformities to allow the generation of *patient-specific *models of walking.**

There is also considerable debate at present about the validity of these techniques (the simulations, the estimations of muscle forces and the forward modelling). Whilst the general principles are sound the techniques are known to be extremely sensitive to certain aspects of their implementation (and may be sensitive to many more). For example the forward modelling in particular is sensitive to how the interaction between the foot and the floor is modelled with there being no clear consensus as to the most appropriate method for this [74,77,81].

**Implementation of these models must be based on robust techniques being developed to validate models, the first step of this is in rigorous analysis of the sensitivity of models to the assumptions on which they are based.**

## Methods for understanding the effect of intervention

Understanding how to interpret clinical gait analysis data is not itself sufficient to allow selection from amongst treatment options (Table 1). For this it is also necessary to know what effect the available interventions are likely to have on someone's walking pattern. If we had a general theory of walking then it might be possible to develop a theoretical basis for considering the effect of any intervention. For patients whose walking could be modelled using a simulation based on specific musculo-skeletal abnormalities it might be possible to use similar simulations to model what might happen if partial correction of those abnormalities were attempted (obviously full correction would restore normal walking!). The author is unaware of any published work at this level at present.

There are then two methods for understanding the effects of intervention in these patients; clinical research to establish what the actual effect of a given intervention is or using knowledge of the mechanisms of walking to predict the effect of modifying the characteristics of the musculo-skeletal anatomy.

By far the most common approach to date has been the use of clinical research – the comparison of gait patterns before and after a particular intervention [47-55,86-88]. Even so there have been comparatively few studies that have given conclusive findings. Many studies which claim to have done so have quite serious methodological flaws. This is particularly true of research into orthopaedic surgery for children with CP where researchers have used retrospective audits of clinical practice to try and answer specific questions. Many of these studies attempt to make inferences about individual procedures which have only ever been performed as part of a multi-level surgical package [47,49-51,54,55]. It is impossible to tell from these studies which effects are due to the particular procedure being considered and which are due to the overall package. Several studies have attempted to separate out those effects by dividing patients into those who have and those who have not had a particular procedure as part of the overall package of surgery and use methods to compare groups similar to those that would be used for a randomised clinical trial [47,54]. The validity of this approach is questionable, however, because generally the two groups of patients were not similar to start with. Those that had the procedure had it because it was considered that the patient needed it and vice versa. Comparison of the two groups to give insight into the effect of the procedure is thus invalid.

**Perhaps the most challenging field of research for clinical gait analysis is in the design and conduct of prospective clinical trials to ascertain the effects of specific treatments on specific patient groups.**

An alternative to the use of clinical trials is to use knowledge of the mechanisms of walking as a basis for modelling the effect of changing that mechanism. Reports of such studies are now starting to emerge. For example Arnold et al. [84] have reported a subject specific model of a cerebral palsy patient with a stiff knee gait and used it to predict the effect of three different potential interventions. These indicated a preferable intervention and the post-intervention gait data showed at least qualitative agreement with the theoretical predictions.

**Application of such techniques to a wider range of clinical problems represents another exciting sphere of research in clinical gait analysis. It may well be that such techniques are limited to the fairly narrow range of interventions that are based on correction of the mechanisms for very specific aspects of walking but identifying the range of potential applications will be an important part of this process**.

## Competing interests

The author has received research funding from Oxford Metrics Plc (Oxford, UK)
